# Epigallocatechin-3-Gallate Upregulates miR-221 to Inhibit Osteopontin-Dependent Hepatic Fibrosis

**DOI:** 10.1371/journal.pone.0167435

**Published:** 2016-12-09

**Authors:** M. L. Arffa, M. A. Zapf, A. N. Kothari, V. Chang, G. N. Gupta, X. Ding, M. M. Al-Gayyar, W. Syn, N. M. Elsherbiny, P. C. Kuo, Z. Mi

**Affiliations:** 1 Department of Surgery, Loyola University Medical Center, Maywood, IL, United States of America; 2 Department of Biochemistry, Faculty of Pharmacy, Mansoura University, Mansoura, Egypt; 3 Division of Gastroenterology and Hepatology, Medical University of South Carolina, Charleston, South Carolina, United States of America; 4 Section of Gastroenterology, Ralph H Johnson Veteran Affairs Medical Center, Charleston, South Carolina, United States of America; 5 Department of Pharmaceutical Chemistry, University of Tabuk, Tabuk, Saudi Arabia; National Institutes of Health, UNITED STATES

## Abstract

Osteopontin (OPN) promotes hepatic fibrosis, and developing therapies targeting OPN expression in settings of hepatic injury holds promise. The polyphenol epigallocatechin-3-gallate (EGCG), found in high concentrations in green tea, downregulates OPN expression through OPN mRNA degradation, but the mechanism is unknown. Previous work has shown that microRNAs can decrease OPN mRNA levels, and other studies have shown that EGCG modulates the expression of multiple microRNAs. In our study, we first demonstrated that OPN induces hepatic stellate cells to transform into an activated state. We then identified three microRNAs which target OPN mRNA: miR-181a, miR-10b, and miR-221. In vitro results show that EGCG upregulates all three microRNAs, and all three microRNAs are capable of down regulating OPN mRNA when administered alone. Interestingly, only miR-221 is necessary for EGCG-mediated OPN mRNA degradation and miR-221 inhibition reduces the effects of EGCG on cell function. In vivo experiments show that thioacetamide (TAA)-induced cell cytotoxicity upregulates OPN expression; treatment with EGCG blocks the effects of TAA. Furthermore, chronic treatment of EGCG in vivo upregulates all three microRNAs equally, suggesting that in more chronic treatment all three microRNAs are involved in modulating OPN expression. We conclude that in in vitro and in vivo models of TAA-induced hepatic fibrosis, EGCG inhibits OPN-dependent injury and fibrosis. EGCG works primarily by upregulating miR-221 to accelerate OPN degradation. EGCG may therefore have utility as a protective agent in settings of liver injury.

## Introduction

Hepatic fibrosis is the wound-healing response that results from chronic liver injury [1, 2]. Extensive liver fibrosis, and the resulting development of nodules of regenerating hepatocytes, leads to cirrhosis. Cirrhosis is characterized by hepatocellular dysfunction, increased resistance to blood flow, and subsequent portal hypertension, a fatal sequela [3]. Liver cirrhosis is estimated to be the 12^th^ leading cause of death in the world; accounting for 1.021 million deaths based on WHO global estimates, and current treatment options are limited. Current research seeks to develop alternative therapies and identify new molecular targets.

Hepatic fibrosis develops when chronic liver injury activates progenitor cells to repair tissue, but these multipotent cells often become dysfunctional with sustained injury and induce fibrogenic repair and excess cell growth [4, 5]. The hallmark of liver fibrosis is the accumulation of extracellular matrix proteins. This alters the hepatic cytoarchitecture, leading to an obstruction of function [1]. Multiple growth factors and cytokines are associated with driving progenitor cell activation and subsequent hepatic fibrosis [1, 4, 5]. Osteopontin (OPN), a secreted phosphoprotein, is one such cell signaling molecules associated with cell injury and fibrosis [4–7]. Studies have shown that measuring OPN in the plasma of liver disease patients is an effective biomarker for assessing the severity of liver fibrosis [8–11]. Furthermore, it has been demonstrated that hepatocytes act as a major source of OPN, and act in a paracrine role in activating HSCs and increasing collagen-I production [12]. Multiples studies have found that neutralizing OPN abrogates the development of fibrosis [4, 5, 12–15]. In a murine model of thioacetamide (TAA)-induced hepatic fibrosis, OPN-knockout mice demonstrated less hepatic damage and a faster resolution of fibrosis [15]. These findings suggest that targeting OPN could be an effective treatment modality in hepatic fibrosis.

Green tea extract has promise for being a source of new molecular therapies. Green tea, made from the dried leaves of the *Camelia Sinensis* plant, is a popular beverage that has been consumed for thousands of years, and Traditional Chinese Medicine has long touted its ability to aid in disease prevention and treatment [16, 17]. Epidemiological studies have found that green tea consumption is associated with a lower incidence of liver disease [18–20]. Epigallocatechin-3-Gallate (EGCG), is a polyphenol found in high concentrations in green tea and green tea extract, and is the most bioactive and well-studied component of green tea. EGCG has multiple health benefits including the attenuation and prevention of liver fibrosis [16, 21, 22]. In vitro analysis shows that EGCG can abrogate liver fibrosis by inhibiting the proliferation of fibroblasts, reducing collagen deposition, and upregulating the mitochondrial respiratory chain [23]. Outside the spectrum of liver disease, EGCG can attenuate TGF-ß expression in pulmonary fibrosis [24].

In addition, EGCG downregulates OPN expression by decreasing the half-life of OPN mRNA. EGCG-mediated OPN mRNA degradation leads to decreased wound closure [25]. However, the mechanism and the effects of EGCG on OPN in vivo have not been evaluated.

One possible mechanism for EGCG-mediated OPN mRNA degradation is through microRNA (miRNA)-mediated mRNA decay. miR-181a targets OPN mRNA and subsequently downregulates OPN protein expression leading to a suppression of migration and adhesion in HepG2 cells [26]. Notably, microarray studies have shown that EGCG modulates the expression of multiple miRNAs [27–29]. In this current study we focused on three miRNAs: miR-221, miR-181a, and miR-10b, because studies have shown that EGCG modulates the expression of these three miRNAs [27–31]. Furthermore, a review of the miRBase Target Database (Available online: http://www.mirbase.org/) showed that these three miRNAs target OPN mRNA.

In this current study we demonstrated that hepatocyte-derived OPN expression induces hepatic stellate cells (HSCs) into an activated state and then investigated whether miRNAs mediate EGCG-dependent OPN mRNA decay using both in vitro and in vivo toxin-induced liver injury and fibrosis models.

## Materials and Methods

### Cell Culture

HepG2 cell line (ATCC# HB-8065) was obtained from American Type Tissue Collection (ATCC, Manassas, VA), seeded at approximately 1.0 × 10^6^ cells/plate, and grown in standard Eagles Minimum Essential Medium (Corning Cellgro, Mediatech, Inc, Manassas, VA) with 10% fetal bovine serum at 37°C and 5% CO_2_. In the following experiments, cells were treated with EGCG (Sigma Aldrich Inc, Saint Louis, MO) at concentrations of 0.02 μg/mL, 0.2 μg/mL, 2 μg/mL, and 20 μg/mL every 12h for 24h unless otherwise specified.

### Coculture

A 0.4 micron transwell system was used for co-culture experiments, with hepatic stellate LX2 cells seeded on the bottom well and HepG2 cells on the top transwell at a 1:1 ratio (30% confluency) in 6-well culture dishes. Cells were grown in standard Eagles Minimum Essential Medium (Corning Cellgro, Mediatech, Inc, Manassas, VA) with 10% fetal bovine serum at 37°C and 5% CO_2_. Experimental groups received 100 nM of OPN-R3 aptamer (APT) or mutant aptamer (MuAPT) daily. Cells were cocultured for a total of 72 hours, after which HepG2 cells were removed and RNA from LX2 cells was acquired using Trizol.

The pharmacologic properties and sequences of OPN-R3 aptamer (APT) and mutant aptamer (MuAPT) have been previously published [32].

### Quantitative Reverse-Transcription Polymerase Chain Reaction (qRT-PCR)

Total RNA including miRNA was isolated from either cultured cell by Trizol (Life Technologies, Carlsbad, CA) or rat liver FFPE tissues by using miRNeasy FFPE kit (Qiagen, Hilden, Germany). To enhance RNA extraction from Trizol, modifications to the manufacturer’s guidelines were performed as previously described [33]. First strand cDNA was synthesized from 1.0 μg of total RNA using the iScript Select cDNA Synthesis Kit (Bio-Rad Laboratories, Hercules, CA) with 250nM of gene specific primers (Integrated DNA Technologies (IDT), San Diego, CA) according to the manufacturer’s instructions. Quantitative real-time PCR was performed with iQ SYBR Green super mix, using the iCycler iQ Real time PCR Detection System (Bio-Rad), according to the manufacturer’s instructions. U6 RNA was used as an endogenous control, and ΔΔC_T_ values were calculated after U6 normalization. PrimeTime qPCR oligonucleotide primers (IDT San Diego, CA) were used for this reaction and their sequences were as follows: U6 Forward: 5’-TGCGGGTGCTCGCTTCGGCAGC-3’, U6 Reverse: 5’-CCAGTGCAGGGTCCGAGGT-3’ [34], miR-221 Primer 1: 5’-CCTGAAACCCAGCAGACAA-3’, Primer 2: 5’-CAGGTCTGGGGCATGAAC-3’; miR-181a Primer 1: 5’-TGGAGTAGATGATGGTTAGCC-3’, Primer 2: 5’-GAGTTTTGAGGTTGCTTGCTTCAGTG-3’; miR-10b Primer 1: 5’-GCATCGACCATATATTCCCCTA-3’, Primer 2: 5’-CAGAGGTTGTAACGTTGTCT-3’

α-SMA: 5’-TAGCACCCAGCACCATGAAGAT-3’; 5’-GAAGCATTTGCGGTGGACAATG-3’ Tenascin-C: 5’-AGCATCACCCTGGAATGGAGGA-3’; 5’-TGTGGCTTGTTGGCTCTTTGGA-3’ Vimentin: 5'-AGAACGTGCAGGAGGCAGAAGAAT-3'; 5'-TTCCATTTCACGCATCTGGCGTTC-3'

### miRNA Transfection

The following miRNA mimics and inhibitors were purchased from Bioneer (Daejeon, Korea): hsa-miR-221-3p mimic, hsa-miR-221-3p inhibitor, hsa-miR-181a-5p mimic, hsa-miR-181a-5p inhibitor, hsa-miR-10b mimic, hsa-miR-10b inhibitor, miRNA mimic Negative control, and miRNA inhibitor Negative control. miRNA mimics and miRNA inhibitors were transfected into cultured cells using Lipofectamine 2000 (Sigma Aldrich Inc) following manufacturer’s protocol.

### Enzyme-Linked Immunosorbent Assay (ELISA)

HepG2 cells were seeded at a density of 3x10^5^ cells per well and incubated overnight. Cells were then treated with either 2μg/ml of EGCG and transfected with miRNA inhibitors or transfected with miRNA mimics alone (see “miRNA Transfection”). After 48h of treatment, cell media was collected and serum OPN protein quantification was performed using a human OPN Quantikine ELISA kit, following the manufacturer’s guidelines (R&D Systems).

### In Vitro Cell Migration Assay

Cell migration assays were performed as previously described [24] with the following modifications. Cells were pretreated for 24h with either 2μg/ml of EGCG and transfected with miRNA inhibitors or transfected with miRNA mimics alone (see “*miRNA Transfection*”). EGCG treated cells continued to receive EGCG 2 μg/ml treatment throughout the experiment including the 48h following the scratch wound. After 24h of pretreatment a pipette tip was used to make a wound and images were taken at 0h, 24h, and 48h. ImageJ (National Institutes of Health) was used to calculate the percent of wound closure.

### Animals and Treatment Outlines

Rat tissue from previous experiments performed by Darweish et al [35] was donated for our experiments. The protocol from those experiments are briefly stated here: Male Sprague Dawely rats weighing 180–200 g were maintained under standard conditions of temperature 25°C, with regular 12 h light/12 h dark cycle and allowed free access to food and water. Rats were classified into the following groups with 10 rats in each group: **Control group:** Rats received intraperitoneal (ip) injection of phosphate buffer saline (PBS, 10 mM, pH 7.4) and served as negative control throughout the study. **Green tea treated control group:** Rats received ip injection of 20 mg/kg green tea (Zhejiang Yixin Pharmaceutical Co., China) dissolved in PBS (10 mM, pH 7.4) twice per week for 16 weeks. **Thioacetamide-treated group:** Rats were injected with thioacetamide (Tocris, Bristol, UK) at a dose of 200 mg/kg, ip twice per week for 16 weeks. **Combined thioacetamide-green tea treated group:** Rats received EGCG (20 mg/kg, ip) twice per week throughout the whole study and were injected with thioacetamide (200 mg/kg, ip) twice per week for 16 weeks starting from the second week after EGCG.

The doses and time course of experiments used for EGCG in this study were in the range of those used in other studies applied for the same animal species [35].

### Immunohistochemistry

Rat liver tissues immunohistochemistry staining were performed, imaged and evaluated by Loyola University Chicago pathology core facility. Primary antibodies used were: OPN (Santa Cruz Biotechnology; sc-21742; 1:200); ki67 (Abram; ab16667; 1:100); p53 (Santa Cruz Biotechnology; sc-6243; 1:200); α-SMA (Santa Cruz Biotechnology, sc-53015; 1:200); TGF-β (Santa Cruz Biotechnology, sc-31608; 1:200)

### Statistical Analysis

Statistical analyses were performed using GraphPad PRIZM (GraphPad Software, San Diego, CA). Paired student’s T-test was used to perform statistical analysis except for analysis of PCR results from coculture experiments in which p values were calculated with one-way ANOVA with bonerroni correction. Results were deemed significant at a confidence interval >95%.

## Results

### OPN induces HSCs to transform into an activated state

**Fig 1:**
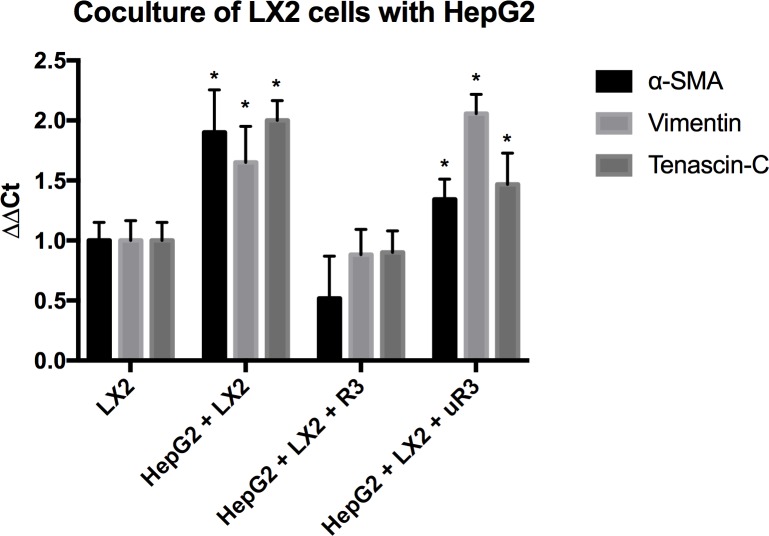
Coculture of LX2 Cells with HepG2. Coculture of LX2 HSCs with high-OPN secreting HepG2 cells induced activation of HSCs while blockade of OPN via APT reverses these effects. Data are presented as mean ± standard deviation of the mean. Coculture of LX2 cells with HepG2 cells caused a significant increase in expression of α-SMA, vimentin, and tenascin-c (all *P*<0.05) while blockade of OPN expression via APT reversed this effect and expression of all three markers are not significant from LX2 controls (*P*>0.05). MuAPT did not reverse the effects of HepG2 coculture, and expression of all three markers were significantly increased from LX2 controls (*P*<0.05). ΔΔCT values were defined using β-actin normalization. *n* = 3, *P<0.05

To demonstrate that OPN activates HSCs, a coculture experiment was performed. The HSC cell line, LX2, was cocultured with HepG2 cells, a high OPN expressing cell line for 72 hours after which HepG2 cells were removed and RNA expression of LX2 cells was measured with qRT-PCR (Fig 1). Coculture of LX2 cells with HepG2 cells caused a ~1.9-fold increase in α-SMA, a ~1.6-fold increase in vimentin, and a ~2-fold increase in tenascin-C (*P*<0.05). Experimental groups that received daily APT demonstrated a reversal of the effects of coculture and the expression of α-SMA, vimentin, and tenascin-c were not significantly different from the LX2 control group (*P*>0.05). Meanwhile, introduction of daily muAPT demonstrated a ~1.3-fold increase in α-SMA, a ~2.1-fold increase in vimentin, and a ~1.4-fold increase in tenascin-C (*P*<0.05).

### EGCG upregulates the expression of target miRNAs in a dose-dependent fashion, but only miR-221 is necessary for EGCG-mediated OPN mRNA degradation

**Fig 2:**
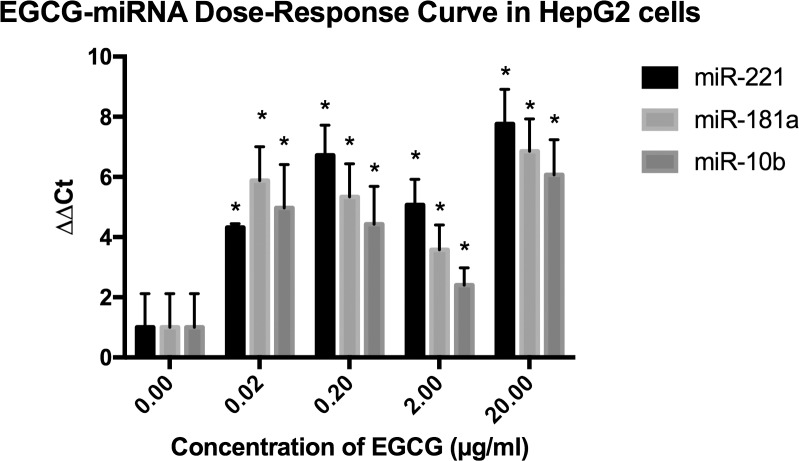
EGCG-miRNA Dose-Response in HepG2 Cells. EGCG increased expression of miR-221, miR-181a, and miR-10b. Data are presented as mean ± standard deviation of the mean. MiR-221, miR-181a, and miR-10b levels are all significantly higher in HepG2 cells treated with 0.02–20 μg/ml EGCG vs non-treated HepG2 cells (all *P*< 0.05). ΔΔCT values were defined using U6 RNA normalization. *n* = 3, *P<0.05.

To demonstrate that EGCG upregulates the expression of miRNAs 221, 181a, and 10b, an EGCG dose-response curve was generated. HepG2 cells were treated with varying concentrations of EGCG (0.02–20μg/ml) for 24 hours before measuring miRNA expression with qRT-PCR (Fig 2). Treatment of HepG2 cells with EGCG at concentrations of 0.02–20 μg/ml caused an upregulation of the expression of all three miRNAs (*p*<0.05). Treatment of 0.02μg/ml EGCG caused a ~10-fold increase in miR-221 expression, a ~30-fold increase in miR-181a, and a ~16-fold increase in miR-10b. Treatment at 0.2μg/ml EGCG caused a ~53-fold increase in miR-221 expression, a ~20-fold increase in miR-181a expression, and a ~11-fold increase in miR-10b expression. Treatment at 2μg/ml EGCG caused a ~17-fold increase in miR-221 expression, a ~6-fold increase in miR-181a expression, and a ~2.6-fold increase in miR-10b expression. Treatment at 20μg/ml EGCG caused a ~109-fold increase in miR-221 expression, a ~58-fold increase in miR-181a expression, and a ~34-fold increase in miR-10b expression. These results demonstrate the ability of EGCG treatment at concentrations of 0.02μg/ml, 0.2 μg/ml, 2μg/ml, and 20μg/ml to upregulate miR-221, miR-181a, and miR-10b expression in HepG2 cells.

**Fig 3:**
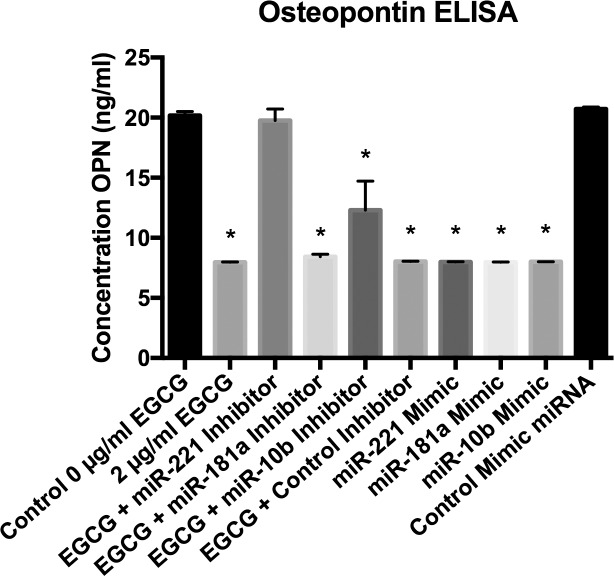
Osteopontin ELISA. EGCG decreased OPN protein levels, meanwhile inhibition of miR-221 reversed the effects of EGCG. Data are presented as mean ± standard deviation of the mean. Treatment with 2μg/ml of EGCG caused a significant ~2.5-fold decrease in OPN protein levels compared with no treatment control, and only inhibition of miR-221 abrogated these effects (7.96 ng/ml ± 0.03 ng/ml vs 20.17ng/ml ± 0.32ng/ml; 19.76 ng/ml ± 0.95 ng/ml; all *P*<0.01). EGCG + miR-221 inhibitor group was not significant from the non-treatment group. Administration of mimics for miR-221, miR-181a, and miR-10b all significantly decreased OPN protein levels (*P<*0.01). *n* = 3.

Next, to assess the role of miRNAs 221, 181a, and 10b in EGCG-mediated OPN protein downregulation, HepG2 cells were treated with 2μg/ml EGCG and 20 pmol of miRNA inhibitors for all three miRNAs or solely with miRNA mimics. After 48h OPN protein expression was measured (Fig 3). The mean concentration of OPN protein in non-treated HepG2 cells expressed was 20.2 ng/ml, whereas treatment of 2μg/ml EGCG significantly reduced OPN protein to 8.0 ng/ml (*p*<0.01). Transfection with all three mimic miRNAs caused a significant reduction in OPN expression (*p*<0.01); only inhibition of miR-221 caused a significant reversal of the effects of EGCG as compared to controls (*p*<0.01). Mean OPN protein concentration for mimic transfection control was 20.5 ng/ml, and not significantly different from control (*p*>0.01). Administration of mimic miR-221, miR-181a, and miR-10b all caused a reduction of OPN protein as compared to transfection control (7.9 ng/ml, 8.0ng/ml, and 7.9 ng/ml, respectively; all *p*<0.01). Transfection of miRNA inhibitor control had no significant effect on OPN protein expression with concomitant 2μg/ml EGCG treatment (8.0 ng/ml; *p*>0.01). Only inhibition of miR-221 significantly reversed the effects of EGCG (19.8 ng/ml; *p*<0.01). Mean OPN protein concentration for miR-181a inhibitor transfection was 8.4 ng/ml and mean OPN protein concentration for miR-10b inhibitor transfection was 12.29 ng/ml; neither were significantly different from control (*p*>0.01).

### EGCG requires miR-221 to decrease wound closure

**Fig 4:**
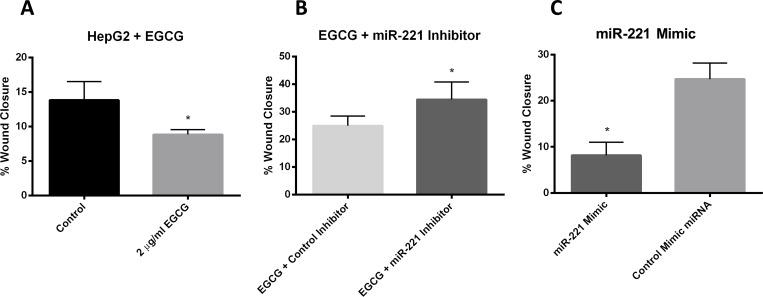
In vitro cell migration assay in HepG2 cells. Data are presented as mean ± standard deviation of the mean. (A) HepG2 cells were pretreated with EGCG (2μg/ml) for 24 hours before scratch was made. (B and C) HepG2 cells were pretreated with EGCG (2μg/ml) for 24 hours with miRNA inhibitors (B) or solely with miRNA mimics (C) before before scratch was made. For all experiments, EGCG treatment was continued throughout. Cells were imaged at 0, 24, and 48 hours after the scratch and migration was quantified based on the percentage of wound healed relative to 0h. *n* = 4, *P<0.05.

To demonstrate that miR-221 expression is necessary for the functional effects of EGCG, we performed a cell migration assay (Fig 4). EGCG treatment decreased cell migration in HepG2 cells after 48 hours (8.8 ± 0.7% vs 13.8 ± 2.7%, *p*<0.05). Inhibition of miR-221 blocked the effects of EGCG: HepG2 cells treated with EGCG and transfected with control miRNA inhibitor had a mean wound closure of 26.4 ± 4.1%, whereas transfection of miR-221 significantly increased wound closure (38.6 ± 4.6%, *p*<0.05). HepG2 cells transfected with control mimic miRNA had a mean wound closure of 32.3 ± 0.92%, while wound closure in HepG2 Cells transfected with mimic miR-221 was significantly reduced (8.2 ± 2.8%, *p*<0.05).

### EGCG Abrogates TAA-Mediated Hepatic Fibrosis and upregulates miR-221

**Fig 5:**
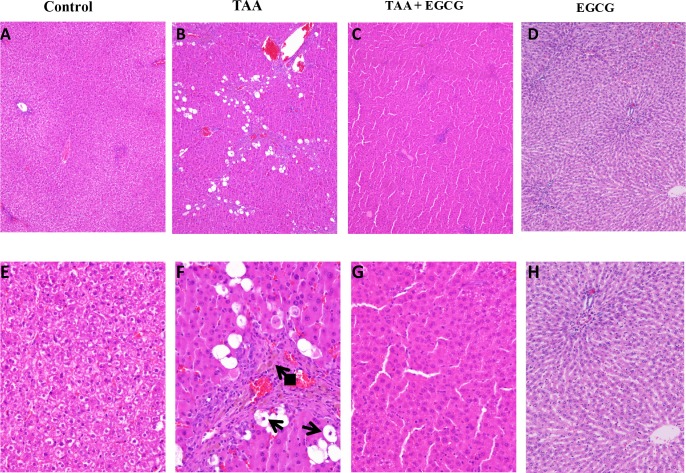
H&E Stain of Rat Liver. In comparison with the normal control and EGCG groups (A and E, D and H, respectively), The TAA treatment group (B and F) shows mild to moderate chronic portal inflammation with lymphoplasmacytic infiltrate and mild interface activity. Numerous pigmented/ceroid macrophages are present in portal tracts (diamond arrow). Significant macrovesicular steatosis is present in the TAA treatment group characterized by large fat vacuoles in periportal hepatocytes. Steatosis accounts for 15% of hepatic parenchyma. In addition, there is hepatocyte ballooning with Mallory-Denk body formation, which is consistent with steatohepatitis. In contrast, treatment with EGCG completely reverses TAA-induced liver injury. Images A-D: 40x; E-H: 100x

**Fig 6:**
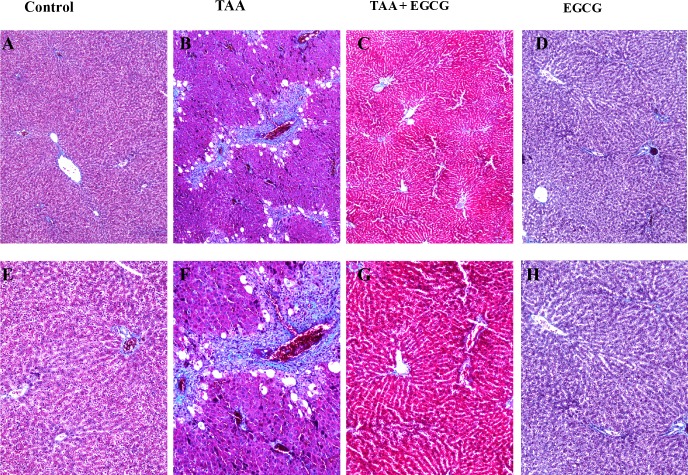
Trichrome Stain of Rat Liver. Trichrome stains show TAA-induced bridging fibrosis. In comparison with control and EGCG groups (A and E, D and H, respectively), the TAA treatment group shows periportal fibrosis with portal to portal fibrotic bridging (B and F); while treatment with EGCG blocks TAA-induced hepatic fibrosis (C and D). Images A-D: 40x; E-H: 100x

**Fig 7:**
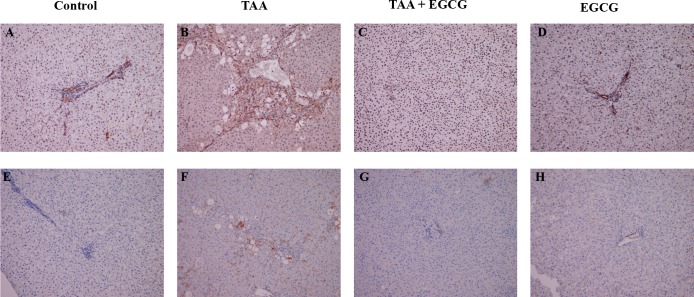
IHC Stains for α-SMA and TGF-β in Rat Liver. Immunohistochemical stains for α-SMA and TGF-β were performed to characterize fibrosis-related gene expression. Control and EGCG groups (A and E, D and H, respectively) demonstrated no staining for α-SMA or TGF-β. TAA treated rat livers showed an upregulation of α-SMA with α-SMA positive cells in the periportal lver parenchyma and portal tract (2+ to 3+ staining) (B). TGF-β IHC showed diffuse, slight increase in expression in TAA treated rat livers (1+ staining) (F). In contrast, livers treated with EGCG and TAA (C and G) demonstrated no expression of either markers. All images: 100x.

Next, to evaluate the effects of EGCG on OPN and EGCG in vivo were conducted using a rat model of toxin-induced fibrosis. Rats were pre-treated for 2 weeks with EGCG. Next, TAA was administered simultaneously with EGCG for 16 weeks and livers were extracted for histological analysis. Hematoxylin and eosin staining of rat liver showed that TAA causes hepatic injury as evidenced by steatosis, Mallory-Denk body formation, and ceroid macrophage infiltration (Fig 5B). The liver of EGCG treated rats, however, showed normal hepatic tissue (Fig 5C). Trichrome staining of hepatic tissue showed that TAA caused significant fibrosis as evidenced by bridging fibrosis in Fig 6B. In contrast, EGCG treatment blocked the effects of TAA, showing normal hepatic tissue (Fig 6C). Next, immunohistochemistry (IHC) stains for α-SMA and TGF-β were performed to characterize fibrosis-related gene expression (Fig 7). TAA-treated hepatic tissue demonstrated an upregulation of both α-SMA (2–3+) and TGF-β (1+) (Fig 7B and 7F, respectively), while EGCG co-treatment blocked the effects of TAA (Fig 7C and 7G, respectively). Control groups (Fig 7A and 7E) and EGCG treatment alone (Fig 7D and 7H) demonstrated no expression of either α-SMA or TGF-β.

**Fig 8:**
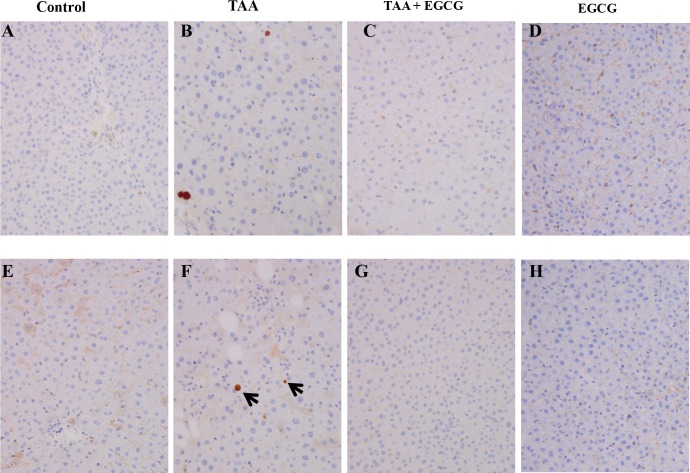
IHC Stains for P53 and Ki67 in Rat Liver. Immunohistochemical stains for P53 and Ki67 were performed to evaluate hepatocyte injury and regenerative changes. The livers in the control group are completely negative for P53 (A) or Ki67 (E) expression. TAA treatment results in upregulation of P53 expression in some hepatocyte nuclei (B) and increases hepatocyte proliferation index as demonstrated by Ki67 nuclear labeling (F). In contrast, no P53 (C) or Ki67 (F) positive cells are identified with TAA + EGCG treatment or with EGCG treatment alone (D and H). All images: 200x.

**Fig 9:**
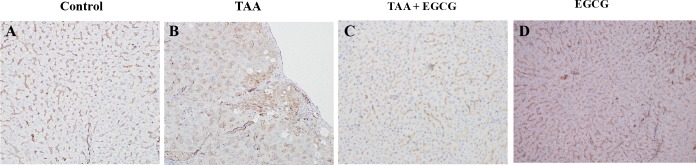
IHC Stain for Osteopontin in Rat Liver. IHC staining for Osteopontin expression was performed. TAA treated rat livers show an upregulation for OPN (B) as compared to control and EGCG treatment groups (A and D). Simultaneous treatment of TAA with EGCG blocks TAA-induced hepatic expression of OPN (C). All images: 200x.

Immunohistochemistry (IHC) stains for p53 were performed to evaluate cellular apoptosis, and revealed that TAA upregulates p53 (Fig 8C) and ki67 (Fig 8D), while EGCG treatment blocked TAA’s effects (Fig 8E and 8F). IHC for OPN demonstrated visible staining within TAA-treated hepatic tissue (2+ expression, Fig 9B). Staining of OPN in the EGCG co-treatment, EGCG alone, and control groups was absent (0–1+ expression, Fig 9A, 9C and 9D).

**Fig 10:**
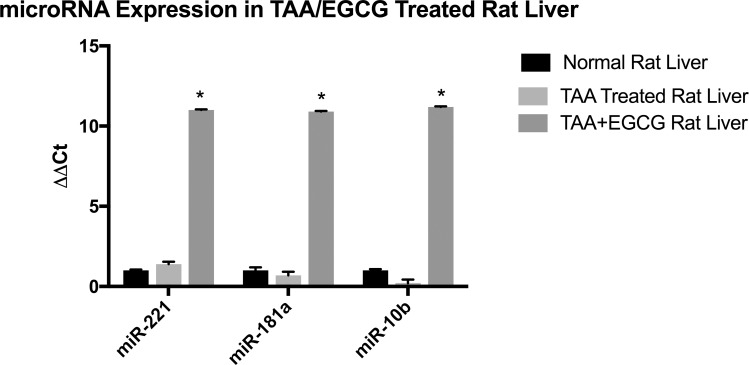
microRNA Expression in TAA/EGCG Treated Rat Liver. EGCG treatment increased miR-221, miR-181a, and miR-10b levels. MicroRNA was extracted from formalin-fixed rat liver tissue using miRNeasy kit (Qiagen). Expression levels of all three miRNAs is significantly greater in livers of rats simultaneously treated with TAA + EGCG vs TAA and control groups. *N* = 3, *P*<0.05.

Finally, MicroRNA was extracted from rat liver tissue and qRT-PCR was performed to measure expression of miR-221, miR-181a, and miR-10b in relation to EGCG treatment (Fig 10). TAA treatment did not cause a significant change in the expression of miR-221, miR-181a, or miR-10b, whereas treatment simultaneous treatment of EGCG with TAA caused a ~1024-fold increase in miR-221, a ~955-fold increase in miR-181a, and a ~1176-fold increase in miR-10b.

## Discussion

EGCG has multiple preventative and therapeutic benefits across a broad scope of disease models. However, before utilizing EGCG in a clinical setting it is important to understand its biological mechanisms. Our work first demonstrated that hepatocyte-derived OPN can activate HSCs and then we elucidated a novel mechanism in which EGCG modulated miRNA expression to target OPN mRNA for degradation and subsequently prevented hepatic fibrosis. Since previous work has shown that OPN expression is upregulated in response to injury [5, 36], we showed that coculturing HSCs with a high-OPN expressing cell-type activated HSCs as indicated by the increased expression of α-SMA, vimentin, and tenascin-C. Furthermore, we demonstrated that OPN paracrine signaling is necessary for HSC activation, as shown in previous work. Since HSCs are a downstream target of OPN protein and do not produce OPN protein or mRNA, we did not measure the effects of EGCG on HSC miRNA expression. Instead, our work focused on the expression of OPN in hepatocytes that express high levels of OPN. In studying these hepatocytes, we also showed that EGCG treatment upregulated the expression of miR-221, miR-181a, and miR-10b at all treatment levels (0.02μg/ml-20μg/ml), but only miR-221 was necessary for EGCG’s effects on OPN expression and cell function in vitro. Interestingly, at doses greater than 0.02 μg/ml, miR-221 exhibited higher levels of expression compared to miRNAs 181a and 10b, but was not significant. Given these findings, further downstream experiments focused on the concentration of 2 μg/ml because previous studies have shown that this concentration is achievable in the plasma of healthy human volunteers who ingest purified green tea extract [37–38]. We next demonstrated that miR-221, miR-181a, and miR-10b are capable of downregulating OPN protein expression with exogenous mimic miRNA transfection, but only inhibition of miR-221 abrogated the effects of EGCG, suggesting that only miR-221 is necessary for EGCG-mediated downregulation of OPN mRNA. To demonstrate that miR-221 is involved in the modulation of fibrotic characteristics, we performed a cell migration assay to assess wound closure. Wound closure and cell migration is associated with fibrosis and is accelerated by OPN expression, and OPN inhibition can delay or even prevent fibrosis [5, 36, 39]. Our cell migration assay demonstrated that miR-221 is necessary for EGCG-induced delay in wound closure, and miR-221 can mimic the effects of EGCG. Previous work from our lab has demonstrated that EGCG treatment at these concentrations does not affect cell viability [25], and therefore should have minimal consequences on wound closure. Alamar Blue bioassay also demonstrated that miR-221 modulation does not affect cell viability when concomitantly treated with TAA with or without EGCG (S1 Fig). Together these results show that miR-221 is necessary for EGCG’s anti-fibrotic properties.

In vivo experiments demonstrated that chronic EGCG treatment can negate the effects of the hepatotoxin TAA. Previous work shows that EGCG can reverse the effects of TAA in rats [35]. Using rat tissue from this previous work we showed that chronic TAA treatment caused hepatic fibrosis (as demonstrated by IHC stains for α-SMA and TGF-β), upregulation of OPN, and upregulation of markers of cell degradation and proliferation (p53 and ki67, respectively), thus indicating that upregulated OPN expression is a component of hepatic fibrosis. Conversely, simultaneous EGCG treatment reversed the expression of OPN as well as other markers of cell damage and fibrosis. Finally, using qRT-PCR we showed that chronic EGCG treatment upregulated miR-221, miR-181a, and miR-10b to similar levels, which our in vitro studies demonstrated is capable of downregulating OPN expression. These results therefore show that miR-221 is necessary for EGCG-mediated OPN mRNA degradation and that long term EGCG treatment can reverse the hepatotoxic and profibrotic effects of TAA.

Studies have shown that miR-221 has multiple effects depending on tissue location and tissue type. In a model of androgen sensitive prostate cancer, researchers found that inhibition of miR-221 in LnCaP cells reduces cell migration [40]. Another study found that miR-221 expression is downregulated in a subset of gastrointestinal stromal tumors, allowing for expression of Kit, and that exogenous miR-221 could act as a putative new therapeutic agent for silencing Kit in these tumors [41]. Research by Felli et al [42] confirm this finding, showing that miR-221 decreases Kit expression in kit-positive leukemic cells.

Importantly, recent findings have also shown that miR-221 expression is critical for hepatocyte survival following injury. In a study of patients with acute liver failure, those who spontaneously recovered had higher serum levels of miR-221 compared to non-recovered patients [43]. Another study found that miR-221 protects hepatocytes from apoptosis in part by regulating p53 upregulated modulator of apoptosis (PUMA) and delays fulminant liver failure in mice [44]. It was also shown that miR-221 accelerates hepatocyte proliferation by regulating the expression of *Arnt* [45]. This research supports our current findings that upregulated miR-221 expression is associated with decreased hepatic damage.

It is also important to note that miR-221 is also associated with tumorigenesis. A meta-analysis of microarray studies found that miR-221 was consistently overexpressed in human hepatocellular carcinoma (HCC) [46], and long term exposure to miR-221 in mice can promote HCC development [47]. Hence, further research should explore the role of miR-221 in hepatic fibrosis and HCC and strategies utilizing miR-221 in settings of liver injury should be careful to balance miR-221’s hepatoprotective effects with its tumorigenic effects.

**Fig 11:**
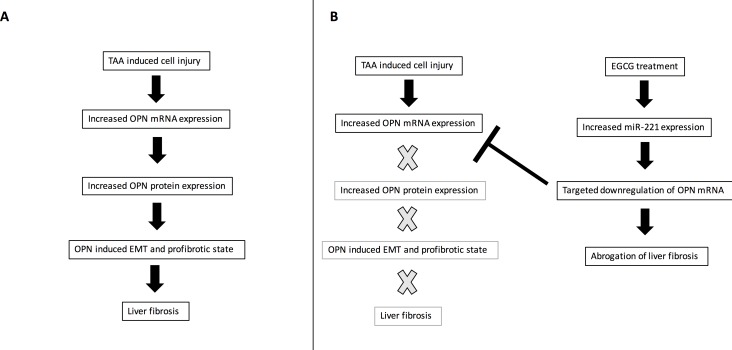
Schematic Representation of EGCG-Mediated Downregulation. Schematic represenation of EGCG-mediated downregulation of OPN protein and prevention of liver fibrosis. TAA-mediated cell damage induces the expression of OPN mRNA and protein which subsequently causes EMT and induction of a profibrotic state (A). However, EGCG treatment abrogates hepatic fibrosis by upregulating the expression of miR-221, which neutralizes OPN mRNA, preventing the downstream effects of OPN expression (B). Black arrows indicate pathway is activated, grey X’s indicate pathway is muted.

Multiple studies have shown that OPN is involved in hepatic fibrosis and that neutralization of OPN is effective in abrogating fibrosis [4,5, 12–15]. Our previous work demonstrated that EGCG can decrease OPN expression through OPN mRNA degradation. And other studies have demonstrated EGCG’s ability to prevent and attenuate hepatic fibrosis [16, 21, 22]. In our current study, we demonstrated that OPN induces HSCs into an activated state. We then found that miR-221 expression is necessary for the antifibrotic characteristics of EGCG as well as for the downregulation of OPN mRNA. miR-221 is therefore critical for recovery from hepatic injury, and its expression can be induced with EGCG treatment. A schematic of this mechanism is demonstrated in Fig 11.

MiRNAs play an intricate role in numerous diseases, and in vivo studies have evaluated the efficacy of miRNA transfection in disease models [48, 49]. Despite these promising results, miRNA therapy is still under development and will require further research before utilization. Meanwhile, green tea and green tea extracts high in EGCG are widely available. Multiple studies have shown that EGCG reduces hepatic fibrosis in multiple in vivo models of hepatic fibrosis [50–52]. Phase I trials have shown that ingestion of green tea tablets produces no adverse effects and with administration 800mg tablets twice a day, it is capable to achieve a plasma concentration used in our in vitro experiments [37–38]. Additionally, analysis of media LDH demonstrated increased cell injury with TAA that is significantly ameliorated in the presence of EGCG (S2 Fig).

There are limitations to the generalizability of our research that further experiments could investigate. HepG2 cells are an in vitro hepatocellular carcinoma (HCC) cell line and thus are not a suitable analogue to cellular changes seen in hepatic injury and fibrosis. In addition, HepG2 cells are commonly used for assessing the effects of new treatment modalities on hepatic cells. Another limitation to our study is that we only assessed one mechanism of EGCG’s effects. It is well known that EGCG can epigenetically modify protein expression through interaction with DNA methyltransferase [53] and that OPN expression can be modified through this same modality [54]. Furthermore, in vivo analysis did not directly assess the effects of miRNAs on OPN expression and hepatic fibrosis. In further studies we seek to generate rat models to further evaluate the interaction between miR-221 and OPN mRNA.

It is also important to note that the mechanism by which EGCG modulates miR-221 expression is unknown. One possible mechanism may be through EGCG interaction with an extracellular receptor. It has been shown that EGCG can interact with the 67-kDa laminin receptor and affect miRNA expression [31]. Interestingly, studies have also shown that EGCG can bind to both DNA and RNA molecules [55, 56]. Using ^1^H NMR spectroscopy, one study found that EGCG modulates miR-122 and miR-33 by direct binding to the miRNAs [57]. Together these findings suggest multiple possible mechanisms by which EGCG can modulate miRNA expression.

In summary, we provided evidence that EGCG treatment upregulates the expression of miR-221, miR-181a, and miR-10b. Functionally, EGCG requires the expression of miR-221 to downregulate OPN protein and its associated fibrogenic properties. In vivo, EGCG treatment is capable of preventing toxin-induced fibrosis, which involves the suppression of OPN expression and the upregulation of miR-221, miR-181a, and miR-10b. EGCG is a safe, accessible, and relatively inexpensive chemical. Understanding it’s mechanism of action allows for better assessment for which disease processes it will have therapeutic potential. Controlling fibrosis and preventing progression of hepatic fibrosis is important for preventing the development of sequelae such as cirrhosis and hepatocellular carcinoma [3–5]. Given our results, EGCG therapy for patients with hepatic fibrosis and the prevention of disease progression proves promising.

## Supporting Information

S1 Fig Cell Viability Assay. To demonstrate that antagomir administration had no effect on cell viability, an Alamar Blue assay was performed. Data shows that there is no significant difference in fluorescence intensity between any of the groups, indicating no changes on cell proliferation with administration of miR-221 mimics or antagomirs. Concentration of TAA was 45 mmol/L.
(DOCX)

S2 Fig LDH Cell Cytotoxicity Assay. To demonstrate that EGCG treatment reduces the effects of TAA-induced cell cytotoxicity, an LDH cell cytotoxicity assay was performed. Treatment of HepG2 cells with EGCG did not significantly increase cell cytotoxicity, while treatment with 45 mmol/L TAA caused a ~8% increase in cell cytotoxicity (*P*<0.05). Simultaneous treatment with 2 μg/ml of EGCG reduced cell cytotoxicity to ~4%, which was significant (*P*<0.05). * = *P*<0.05, significant difference between control, † = *P*<0.05, significant difference between TAA alone treatment group.
(DOCX)

S1 File Raw Data. Raw data for Figs 1, 2, 3, 4 and 10 have been compressed into a single zip file.
(ZIP)

## Abbreviations

OPN: osteopontin
EGCG: epigallocatechin-3-gallate
TAA: thioacetamide
