# Supplementary material for: Epigallocatechin-3-Gallate Upregulates miR-221 to Inhibit Osteopontin-Dependent Hepatic Fibrosis
Source: PLoS One. 2016 Dec 9;11(12):e0167435. doi: 10.1371/journal.pone.0167435 (PMC5147893; doi:10.1371/journal.pone.0167435)
Supplement: S1 Fig — To demonstrate that antagomir administration had no effect on cell viability, an Alamar Blue assay was performed. Data shows that there is no significant difference in fluorescence intensity between any of the groups, indicating no changes on cell proliferation with administration of miR-221 mimics or antagomirs. Concentration of TAA was 45 mmol/L. (DOCX) [file pone.0167435.s001.docx]

**Supplementary Figure 1.**

**
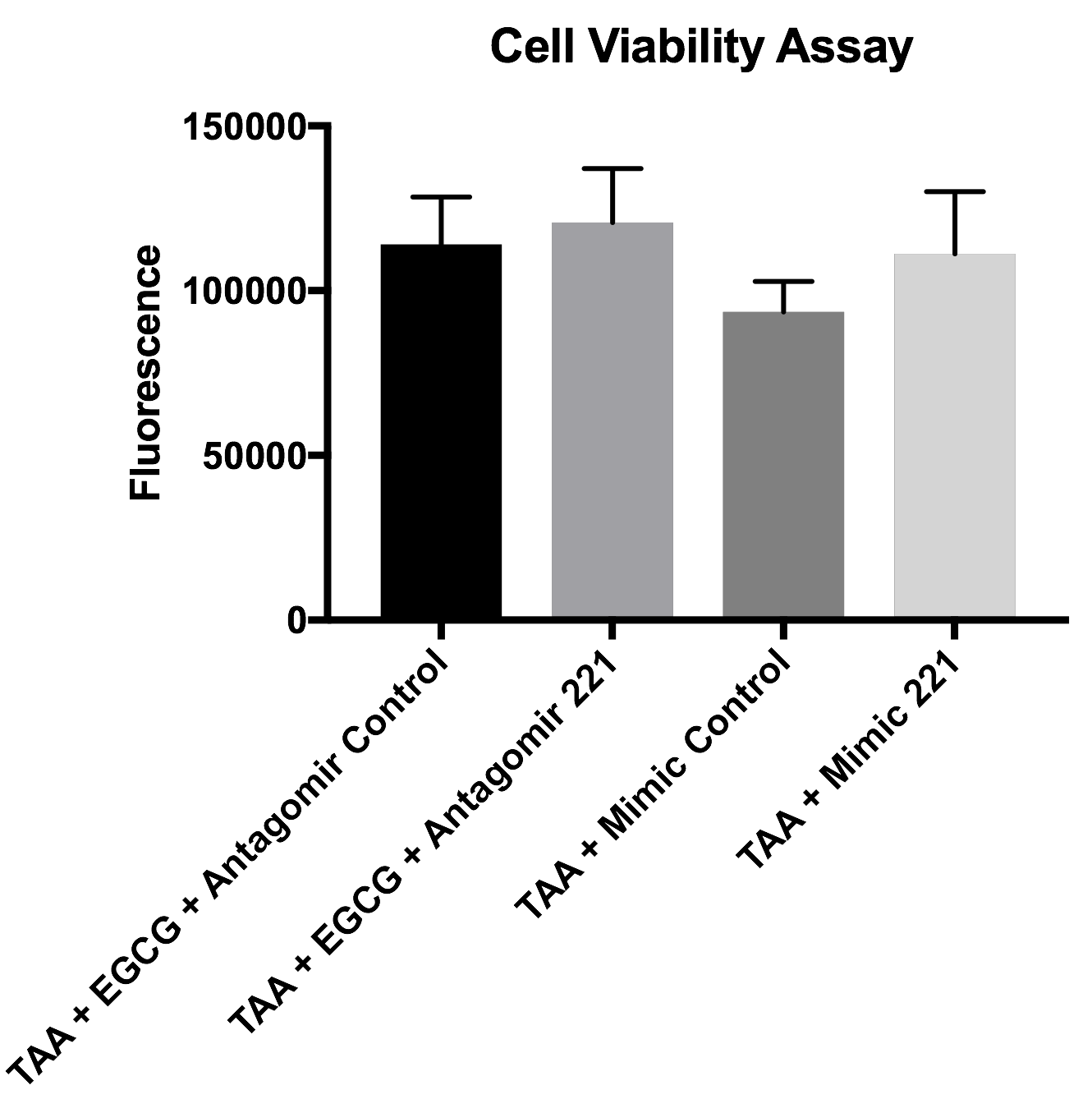
**

**Materials and Methods:**

HepG2 cells were seeded at a density of 1,000 cells/100µL wells and incubated overnight. Cells were then transfected with miRNA mimics and antagonists (see “*miRNA Transfection*”) for 24hours. TAA 45mmol/L and EGCG 2µg/ml were then administered to cells transfected with antagomirs and Alamar Blue assay was performed following manufacturer’s protocol (Thermo Scientific, Rockford, IL). TAA concentration was selected based on published research (1).

**Results:**

To demonstrate that antagomir administration had no effect on cell viability, an Alamar Blue assay was performed. Data shows that there is no significant difference in fluorescence intensity between any of the groups, indicating no changes on cell proliferation with administration of miR-221 mimics or antagomirs. Concentration of TAA was 45 mmol/L.

**Reference:**

1. Stankova P, Kucera O, Lotkova H, Rousar T, Endlicher R, Cervinkova Z. The toxic effect of thioacetamide on rat liver in vitro. Toxicol In Vitro 2010 Dec;24(8):2097-2103.
