# Supplementary material for: Epigallocatechin-3-Gallate Upregulates miR-221 to Inhibit Osteopontin-Dependent Hepatic Fibrosis
Source: PLoS One. 2016 Dec 9;11(12):e0167435. doi: 10.1371/journal.pone.0167435 (PMC5147893; doi:10.1371/journal.pone.0167435)
Supplement: S2 Fig — To demonstrate that EGCG treatment reduces the effects of TAA-induced cell cytotoxicity, an LDH cell cytotoxicity assay was performed. Treatment of HepG2 cells with EGCG did not significantly increase cell cytotoxicity, while treatment with 45 mmol/L TAA caused a ~8% increase in cell cytotoxicity (P<0.05). Simultaneous treatment with 2 μg/ml of EGCG reduced cell cytotoxicity to ~4%, which was significant (P<0.05). * = P<0.05, significant difference between control, † = P<0.05, significant difference between TAA alone treatment group. (DOCX) [file pone.0167435.s002.docx]

**S2 Figure 2**

**
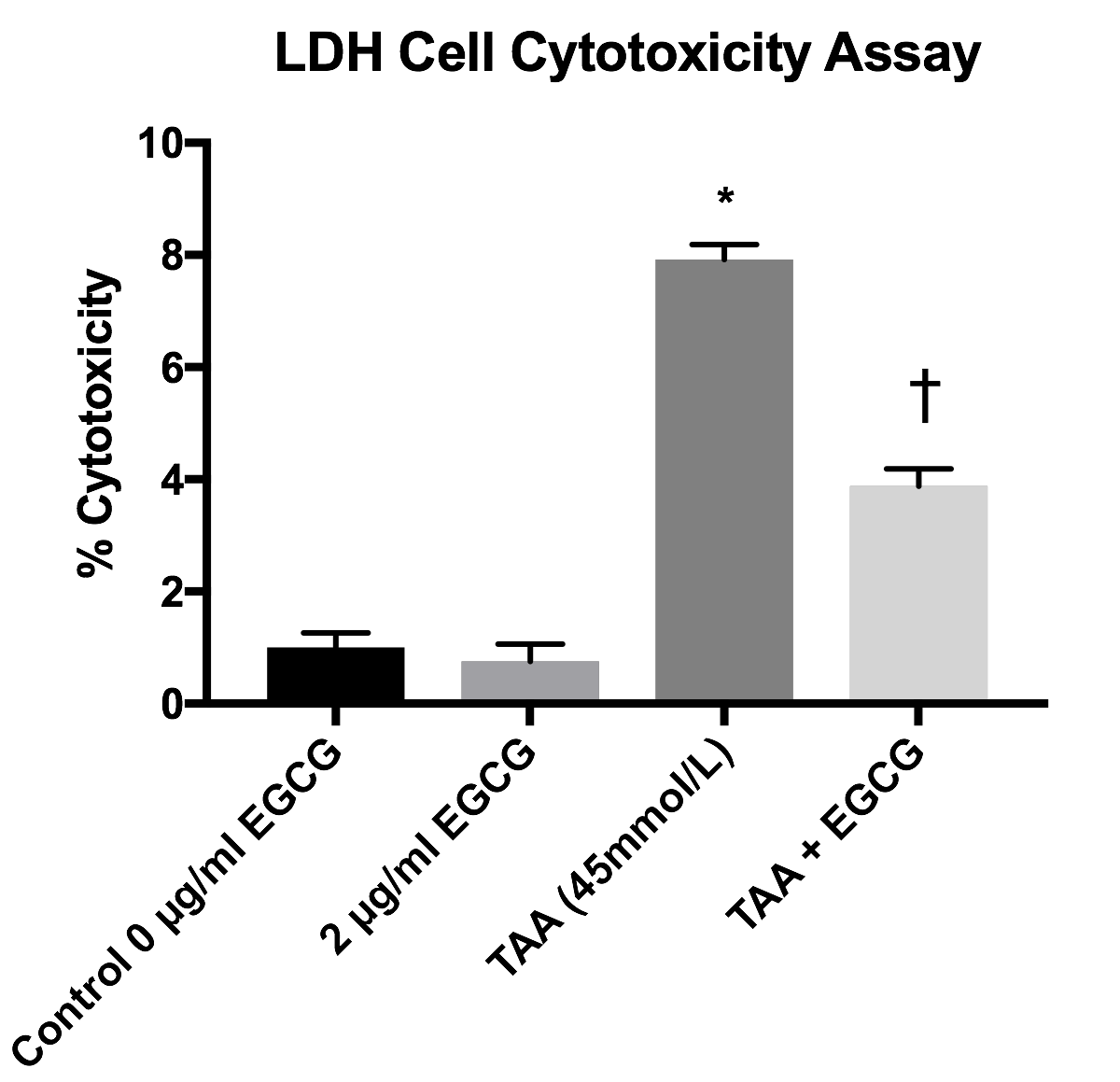
**

**Materials and Methods:**

HepG2 cells were seeded at a density of 4,000 cells per well and incubated overnight. Cells were then treated with TAA 45mmol/L or TAA 45mmol/L with 2 µg/ml EGCG for 24h. Cell media was then collected and LDH assay was performed using Pierce LDH Cytotoxicity Assay Kit (Thermo Scientific, Rockford, IL) following manufacturer’s protocol. TAA concentration was selected based on published research [1].

**Results:**

To demonstrate that EGCG treatment reduces the effects of TAA-induced cell cytotoxicity, an LDH cell cytotoxicity assay was performed. Treatment of HepG2 cells with EGCG did not significantly increase cell cytotoxicity, while treatment with 45 mmol/L TAA caused a ~8% increase in cell cytotoxicity (*P*<0.05). Simultaneous treatment with 2 µg/ml of EGCG reduced cell cytotoxicity to ~4%, which was significant (*P*<0.05). * = *P*<0.05, significant difference between control, † = *P*<0.05, significant difference between TAA alone treatment group.

**Reference:**

1. Stankova P, Kucera O, Lotkova H, Rousar T, Endlicher R, Cervinkova Z. The toxic effect of thioacetamide on rat liver in vitro. Toxicol In Vitro 2010 Dec;24(8):2097-2103.
